# Evaluation of the recurrence pattern of gastric cancer after laparoscopic gastrectomy with D2 lymphadenectomy

**DOI:** 10.1186/s40064-016-2535-4

**Published:** 2016-06-21

**Authors:** Yuichiro Kawamura, Seiji Satoh, Yusuke Umeki, Yoshinori Ishida, Koichi Suda, Ichiro Uyama

**Affiliations:** Division of Upper GI, Department of Surgery, Fujita Health University, 1-98 Dengakugakubo, Kutsukake, Toyoake, Aichi 470-1192 Japan; Department of Surgery, Kokura Memorial Hospital, 3-2-1, Asano, Kokura-Kita, Kitakyusyu, Fukuoka 802-8555 Japan; Department of Surgery, National Hospital Organization Himeji Medical Center, 68, Honmachi, Himeji, Hyogo 670-8520 Japan

**Keywords:** Gastric cancer, Laparoscopic gastrectomy, D2 lymph node dissection, Recurrence pattern

## Abstract

**Background:**

The aim of this study was to analyze the oncological aspects of gastric cancer following laparoscopic gastrectomy with D2 lymphadenectomy (LG-D2).

**Methods:**

We retrospectively evaluated the long-term outcomes of 354 patients who underwent LG-D2 for primary gastric cancer. Recurrence patterns and predictors of peritoneal metastasis were analyzed.

**Results:**

Median follow-up time was 43.8 months. Five-year overall survival rates for yp/pStages I, II, and III gastric cancer were 93.7, 78.5, and 42.2 %, respectively. Recurrence was observed in 86 patients. Peritoneal metastasis was the most frequent recurrence pattern (*n* = 51), followed by hepatic metastasis (*n* = 17). Lymphatic recurrence at distant sites was observed in 10 patients. No locoregional lymph node metastasis or local recurrence was seen. Nine of 51 cases of peritoneal recurrence were detected by probe laparoscopy. Peritoneal recurrence rates were significantly higher in yp/pT4 and yp/pN3 diseases compared with yp/pT ≤ 3 and yp/pN ≤ 2 diseases. Multivariate analyses demonstrated that yp/pT4, yp/pN3, tumor size ≥70 mm, vascular invasion, and undifferentiated tumors were predictors of peritoneal recurrence following LG-D2.

**Conclusion:**

Long-term outcomes of gastric cancer following LG-D2, including recurrence patterns and predictors of peritoneal metastasis, were comparable to those following open D2 gastrectomy. LG-D2 showed good local control. Probe laparoscopy after LG may be effective in detecting peritoneal recurrence, which is not determined with less invasive examinations, including a CT scan. Future large-scale prospective studies are desirable to evaluate not only surgical but also oncological benefits and safety of LG-D2 for advanced gastric cancer.

## Background

The first report of a laparoscopic distal gastrectomy (LDG) for gastric cancer was described by Kitano et al. (1994). A number of randomized controlled trials (Kim et al. 2010; Katai et al. 2010) have reported equivalent short-term outcomes, including the incidence of anastomotic leakage and a pancreatic fistula, following LDG with D1+ lymphadenectomy for ≤cT2N0 gastric cancer to those following an open distal gastrectomy (ODG), at least as long as LDG was performed by an experienced laparoscopic surgeon. Regarding ODG, previous studies (Degiuli et al. 2010; Songun et al. 2010) have reported that D2 lymphadenectomy reduces local recurrence and improves long-term outcomes compared with D1 lymphadenectomy in patients with advanced gastric cancer.

Laparoscopic gastrectomy with D2 lymphadenectomy (LG-D2) is currently performed for the treatment of advanced gastric cancer at some specialized institutions (Huscher et al. 2005; Cai et al. 2011). Concerning short-term outcomes, recent meta-analyses (Zeng et al. 2012; Zou et al. 2014; Huang et al. 2014) have reported no significant difference in the number of harvested lymph nodes (LN) between ODG and LDG. However, detailed studies assessing recurrence patterns and long-term outcomes following LG-D2 are lacking.

In 1999, we reported on the technical aspects of LDG with D2 lymphadenectomy (LDG-D2) and laparoscopic total gastrectomy with D2 lymphadenectomy (LTG-D2) (Uyama et al. 1999a, b) as well as short- and long-term outcomes following LG for gastric cancer (Yoshimura et al. 2011; Shinohara et al. 2013). In this study, we retrospectively analyzed long-term outcomes of 346 gastric cancer patients who underwent LG-D2, especially focusing on the recurrence pattern.

## Methods

### Patients

LG-D2 was performed in 369 consecutive cases of patients with cStage ≥ IB gastric cancer at the Fujita Health University Hospital between August 1997 and December 2011. Preoperative chemotherapy (PC) was performed for 139 of the 369 patients. Of the 369 identified cases, 15 patients were diagnosed with yp/pStage IV of the disease. These 15 yp/p Stage IV patients included three with P0CY1, P1CY0, and H1 and six with M1 gastric cancer, and they were excluded. Consequently, a clinical database containing the data of these 354 patients who underwent an R0 resection by LG-D2 for yp/pStages 0–III gastric cancer were retrospectively reviewed. The pathological cancer stage was determined according to the 14th edition of the Japanese Classification of Gastric Carcinoma (2011a).

### Indication and regimen of preoperative chemotherapy (PC)

After July 2005, Neoadjuvant chemotherapy was used for patients who were diagnosed with ≥cT2 or ≥cN1 gastric cancer and who agreed with a PC treatment. For these patients, S-1 (80 mg/m^2^) was administered from Day 1 to Day 21, CDDP (60 mg/m^2^) was injected on Day 8, and a 2 week washout period was provided. This 5-week regimen was performed twice with 2 more weeks of washout period before surgery. Induction chemotherapy (S-1 80 mg/m^2^ Day 1–14 + CDDP 35 mg/m^2^ Day 8, or Docetaxel 30 mg/m^2^ Day 1, 15 + CDDP 30 mg/m^2^ Day 1, 15 + S-1 80 mg/m^2^ Day 1–14) was used for StageIV disease diagnosed by probe laparoscopy or cytology, and radical gastrectomy was conducted when downstaging was achieved. Although downStaging was achieved for 7 of 10 Stage IV patients, they were excluded as Stage IV because P1 or Cy1 was diagnosed pathologically via staging laparoscopy before chemotherapy.

### Definition of D2 lymphadenectomy

The extent of gastric resection and LN dissection in LDG-D2 and LTG-D2 were determined according to the 2010 Japanese Gastric Cancer Treatment Guidelines (2011b). Accordingly, in LDG-D2, LN stations 1, 3, 4sb, 4d, 5, 6, 7, 8a, 9, 11p, and 12a were dissected. In addition to those LN stations, LN stations 2, 4sa, 10, and 11d were dissected in LTG-D2. Regarding the extent of splenic hilar LN dissection, a D2 lymphadenectomy combined with a distal pancreaticosplenectomy (D2 + PS) was performed in patients with tumors infiltrating into the pancreatic body or tail. A D2 lymphadenectomy combined with a splenectomy (D2 + S) was performed in patients with LN metastasis at station 11d or 10, or in patients with a greater curvature invasion. A spleen-preserving D2 lymphadenectomy (D2-S) was performed in patients with tumor depths ≥cT3 without LN metastasis at station 11d or 10, whereas a D2 lymphadenectomy with preservation of station 10 LNs, and the spleen (D2-10) was performed in patients without a greater curvature invasion and with tumor depths ≤cT2 (Nakauchi et al. 2015).

### Perioperative management, postoperative chemotherapy, and oncologic follow up

Most of our LG perioperative management details have been previously reported (Shinohara et al. 2013; Suda et al. 2015). From 1997 to December 2004, some patients with pStage II/III gastric cancer received adjuvant chemotherapy, but the indication and regimen were not defined during this period. After Jan 2005, patients with yp/pStage II or III cancer received S-1-based adjuvant chemotherapy for 1 year and those with yp/pStage IV cancer received S-1-based definitive chemotherapy according to the 3rd Edition of the Japanese Gastric Cancer Association Guidelines ( 2011b). Patients with ypStage I gastric cancer also received S-1 based adjuvant chemotherapy for 1 year when a Grade ≥ Ib pathological response of PC was observed. Regarding oncologic follow up, the discharged patients visited our outpatient clinic at least after 1 month, 3 months, and then every 6 months until 5 years after surgery. In the outpatient clinic, regular laboratory with carcinoembryonic antigen and carbohydrate antigen 19-9 and physical examinations were performed. The patients were examined by chest and abdominopelvic computed tomographic (CT) scans every 6 months to detect any local recurrence and systemic metastasis. An upper gastrointestinal endoscopy was done every year to detect any local recurrence and metachronous multicentric or multiple cancers. Ultrasonography (US), magnetic resonance imaging (MRI), positron emission tomography-CT (PET-CT), or scintigraphy was used in combination when necessary. Peritoneal metastasis was definitively diagnosed by ascitic cytology or probe laparoscopy.

### Definition of the recurrence pattern and survival time

The recurrence pattern was determined according to the primary recurrence site diagnosed by imaging studies (CT, US, MRI, scintigraphy, or PET-CT), ascitic cytology, or probe laparoscopy. Locoregional LN metastasis was defined as the presence of lymphatic metastases within the D2 lymphadenectomy area. Distant LN metastasis was defined as the presence of any LN metastasis outside the area dissected by a D2 lymphadenectomy. Local recurrence was defined as the presence of non-lymphatic cancer tissue within the surgical area, residual stomach, or anastomotic site. Hematogenous metastasis was defined as the presence of any distant metastasis, except hepatic metastases. In case multiple metastases patterns occurred simultaneously, the dominant metastasis was determined based on the number and size of the metastatic lesions. Recurrence-free survival (RFS) was defined as the period from initial surgery to the first detection of recurrence or whatever was the cause of death. Survival after recurrence (SAR) was defined as the period from the first detection of recurrence to the time of death. Overall survival (OS) was defined as the period from initial surgery to the time of death. Censoring may occur if a patient reached the planned end of the study or was lost to follow-up.

### Multivariate analysis of peritoneal recurrence-free survival

Multivariate analysis of peritoneal recurrence-free survival was performed to determine the predictive factors for peritoneal metastasis. Peritoneal recurrence-free survival was defined as the period from initial surgery to the first detection of peritoneal recurrence or death. Predictive factors evaluated were age (≤64 or ≥65), gender (male or female), body mass index (≤21.0, 21.0–25.0, or >25.0), presence of comorbidities (yes or no), previous operations (yes or no), American Society of Anesthesiologists’ **Classification (Class ≤2 or Class 3), preoperative chemotherapy (yes or no), histological tumor type (differentiated or undifferentiated), tumor size (<70 or ≥70 mm), T factor (≤T3 or T4), N factor (≤N2 or N3), lymphatic invasion (positive or negative), vascular invasion (positive or negative), and operative procedure (LDG or LTG). Each factor was divided into two or three categories.

### Statistical analyses

All statistical analyses were performed using the Statistical Package for Social Sciences (SPSS) version 19.0J for Windows (SPSS Inc., Chicago, Illinois, USA). Independent continuous variables were compared by the Mann–Whitney *U* test or Kruskal–Wallis test, and categorical variables were compared by the χ^2^ (Chi-square) test or Fisher’s exact test. Long-term outcomes were analyzed using Kaplan–Meier methods with the log-rank test and Cox regression. Univariate analyses were performed for all potentially confounding variables and effect modifiers. Considering the relatively small sample size, all variables with a significant level of *p* < 0.05 in the univariate analysis were included as independent variables.

## Results

### Clinical characteristics and survival rates

**Fig. 1:**
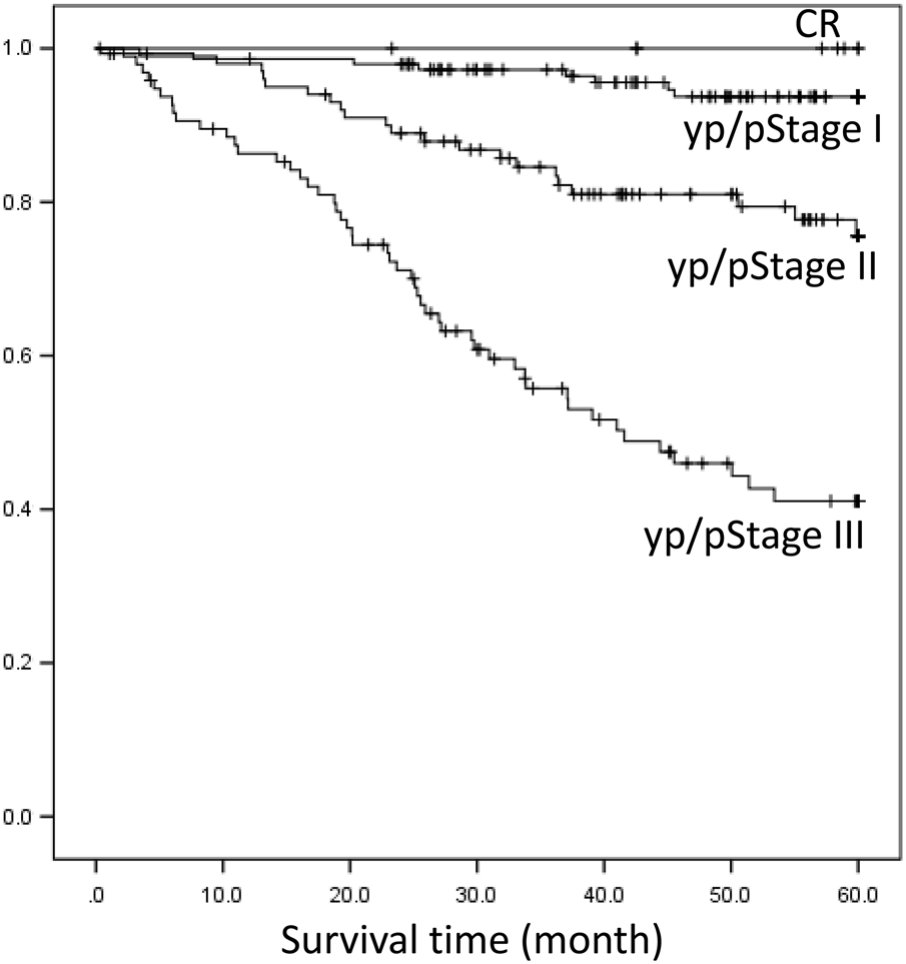
Survival curve according to gastric cancer disease stages. *Stage I*, yp/pstages IA and IB; *Stage II*, yp/pstages IIA and IIB; and *Stage III*, yp/pstages IIIA, IIIB, and IIIC

Clinical characteristics of the 354 analyzed patients are summarized in Table 1. Mean age was 62.6 ± 10.3 years. One or more comorbidities and prior abdominal surgery were identified in 174 patients (49.2 %) and 46 patients (13.0 %), respectively. Preoperative chemotherapy was performed for 128 patients. A total of 251 patients underwent LDG and 103 patients underwent LTG. The proportion of patients who underwent LTG was 29.1 % of all analyzed patients. Of the 103 patients who underwent LTG, splenectomy was performed in 61 patients, of which a combined distal pancreatectomy was performed in 22 patients. Combined resection of the transverse colon to achieve an R0 resection was performed in one patient who underwent LDG and two patients who underwent LTG. The mean tumor size in patients who underwent LTG was significantly greater than in patients who underwent LDG (*p* < 0.001). A pathologically complete response was observed in eight patients who underwent PC. The yp/pT ≥ 2 and yp/pN ≥ 1 diseases were diagnosed in 222 patients (62.7 %) and 154 patients (43.5 %), respectively. The median follow-up period was 43.8 months. During the observation period, 71 patients (20.1 %) died of gastric cancer and eight patients (2.3 %), including one patient (0.3 %) who died due to operative complications, died from other causes. Censoring occurred in 22 patients (6.2 %) within 5 years. OS curves according to the disease stage are shown in Fig. 1. 5-year OS rates stratified by yp/pStage I, II, and III were 93.7, 78.5, and 42.2 %, respectively.

**Table 1:** Clinical characteristics of patients (*n* = 354)

Variables	Patients with PC (n = 128)	Patient without PC (n = 226)	All patients (n = 354)
Age (y/o)^a^	63.0 ± 9.5	62.4 ± 10.8	62.6 ± 10.3
Gender (male:female)	96: 32	148: 78	244: 1110
Body mass index (kg/m^2^)^a^	22.4 ± 3.4	21.9 ± 3.1	22.1 ± 3.2
Comorbidity: *n* (%)	63 (49.2)	111 (49.1)	174 (49.2)
Prior operations: *n* (%)	14 (10.9)	32 (14.2)	46 (13.0)
ASA: *n* (%)			
Class 1	62 (48.4)	110 (48.7)	172 (48.6)
Class 2	50 (39.1)	96 (42.5)	146 (41.2)
Class 3	16 (12.5)	20 (8.8)	36 (10.2)
Tumor size (mm)^a^	48.0 ± 33.0	39.3 ± 24.4	42.4 ± 28.1
Type of resection: *n* (%)			
LDG	74 (57.8)	177 (78.3)	251 (70.9)
LTG	54 (42.2)	49 (21.7)	103 (29.1)
Harvested lymph nodes (*n*)^a^	43.8 ± 15.3	44.6 ± 15.9	44.3 ± 15.7
Pathological status (yp/p): *n* (%)			
CR	8 (6.3)	0 (0)	8 (2.3)
T1	24 (18.8)	100 (44.2)	124 (35.1)
T2	19 (14.8)	40 (17.7)	59 (16.7)
T3	36 (28.1)	27 (11.9)	63 (17.8)
T4	41 (32.0)	59 (26.1)	100 (28.2)
Lymph node metastasis (yp/p): *n* (%)			
N0	70 (54.7)	130 (57.5)	200 (56.5)
N1	20 (15.6)	39 (17.3)	59 (16.7)
N2	20 (15.6)	35 (15.5)	55 (15.5)
N3	18 (14.1)	22 (9.7)	40 (11.3)
Stage (yp/p): *n* (%)			
CR	8 (6.3)	0 (0)	8 (2.3)
Stage I	36 (28.1)	113 (50.0)	149 (42.1)
Stage II	43 (33.6)	58 (25.7)	101 (28.5)
Stage III	41 (32.0)	55 (24.3)	96 (27.1)
Median follow-up period (month)	40.9	45.4	43.8

Values in parentheses are percentages unless otherwise indicated

*PC* preoperative chemotherapy, *CR* pathological complete response to PC, *ASA* American Society of Anesthesiologists, *LDG* laparoscopic distal gastrectomy, *LTG* laparoscopic total gastrectomy

^a^Values expressed as the mean ± SD

### Recurrence patterns, RFS, SAR, and OS

During the follow-up period, gastric cancer recurrence was observed in 86 of the 354 patients (24.3 %). The most frequent recurrence pattern was peritoneal metastasis, which was observed in 51 patients (59.3 %), followed by hepatic metastasis, which was observed in 17 patients (19.8 %). Hematogenous metastasis was observed in eight cases (9.3 %). Distant lymphatic recurrence was observed in 10 cases (11.6 %). Neither locoregional lymphatic recurrence nor local recurrence in the D2 dissection area was observed. In 11 patients, a postoperative probe laparoscopy was performed to confirm peritoneal recurrence. Consequently, in nine cases, peritoneal recurrence was detected by probe laparoscopy as the first recurrence site. The SAR of patients with peritoneal recurrence was significantly shorter than that of patients with distant lymph node recurrence (*p* = 0.012) (Table 2). Multiple metastasis patterns were observed in eight (9.3 %) of the 86 patients at the initial recurrence, each pattern is summarized in Table 3.

**Table 2:** Recurrence patterns and survival times (*n* = 86)

Recurrence pattern	*N*	%	RFS (month)	SAR (month)	OS (month)
Peritoneal metastasis	51	59.3	16.6	6.2	24.8
Hepatic metastasis	17	19.8	9.8	8.1	21.4
Hematogenous metastasis	8	9.3	19.7	11.5	25.6
Bone	(4)				
Ovarium	(2)				
Skin	(1)				
Lung	(1)				
Lymph node metastasis	10	11.6	14.5	18.7	35.0
Distant lymph node	(10)				
Local lymph node	(0)				

*OS* overall survival, *RFS* recurrence-free survival, *SAR* survival after recurrence

**Table 3:** Multiple patterns of metastasis at the initial recurrence (*n* = 8)

Recurrence pattern (dominant site)	Synchronous metastatic site	*N*
Peritoneal metastasis	Distant lymph node	3
	Ovarium	2
	Lung	1
Hepatic metastasis	Brain	1
Lymph node metastasis	Lung	1

**Fig. 2:**
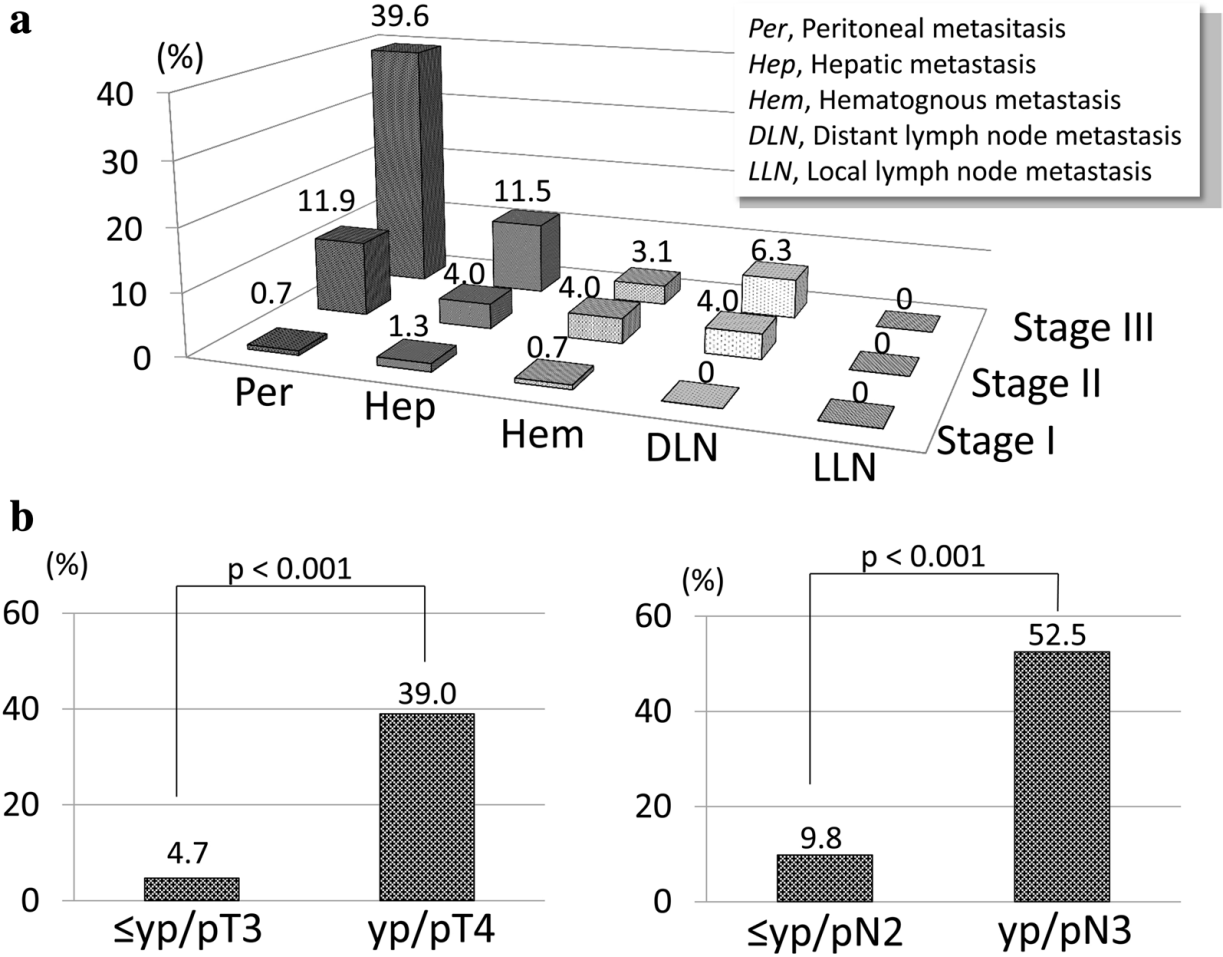
Relationship between recurrence patterns and gastric cancer disease stages. **a** Recurrence rate according to the recurrence pattern and disease stage. The recurrence rate of peritoneal (*p* < 0.001) and hepatic (*p* < 0.001) metastasis increased concurrently with increase in yp/pStage. **b** Comparison of the recurrence rate according to yp/pT and yp/pN factors

The incidences of recurrence according to the disease stage and recurrence pattern are shown in Fig. 2a. The recurrence rate of peritoneal (*p* < 0.001) and hepatic (*p* < 0.001) metastasis was elevated in correspondence with the yp/pStage. Further, the recurrence rate of peritoneal metastasis in yp/pT4 gastric cancer cases was significantly greater than in yp/pT ≤ 3 gastric cancer cases (*p* < 0.001). Additionally, the rate of peritoneal metastasis in yp/pN3 gastric cancer cases was significantly greater than in yp/pN ≤ 2 gastric cancer cases (*p* < 0.001, Fig. 2b).

**Fig. 3:**
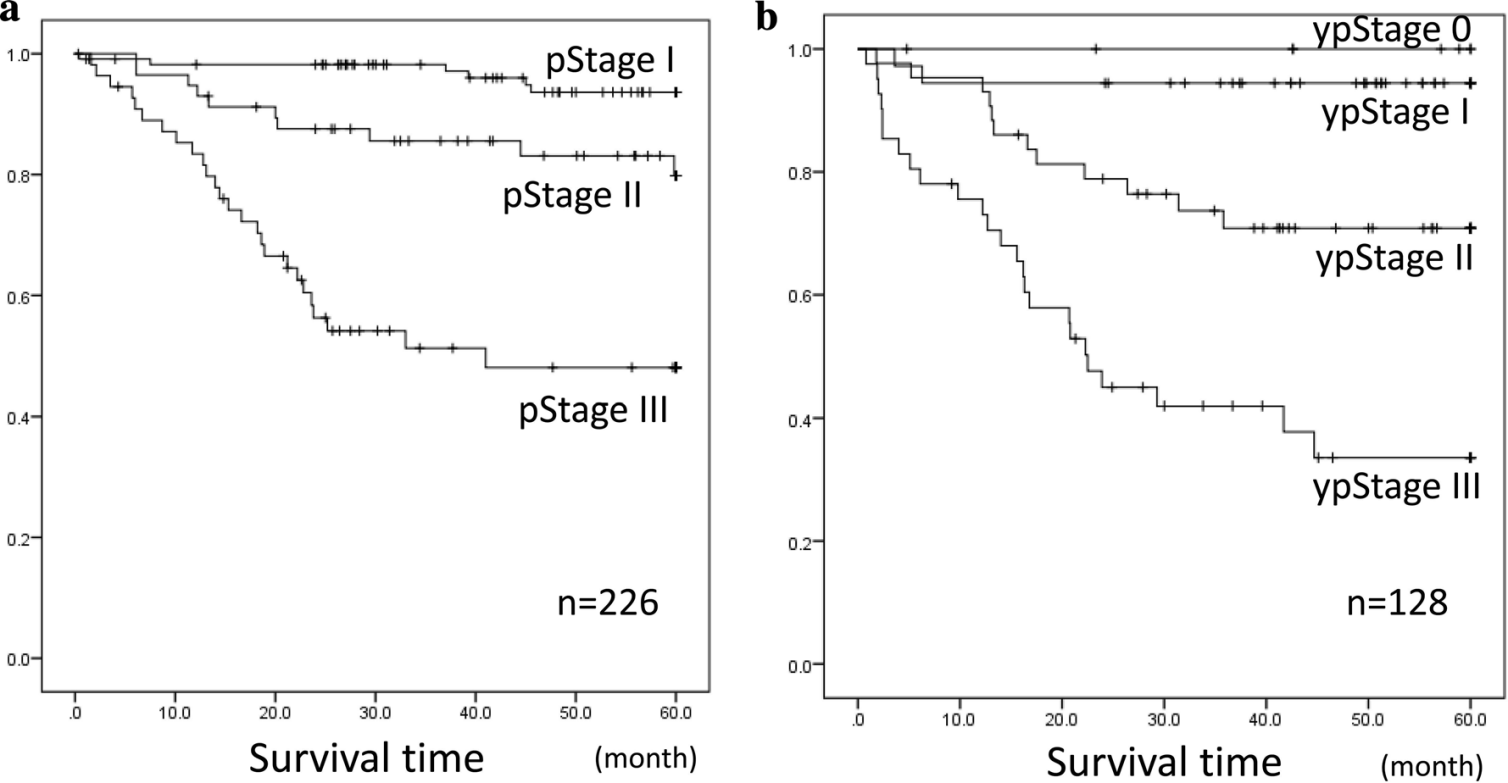
RFS curves according to the yp- and p-Stage. **a** RFS curves according to the p-Stage. **b** RFS curves according to the yp-Stage

RFS curves according to the yp- and p-stage are shown in Fig. 3. Recurrence was not observed in any of the eight patients with a complete pathological response to PC within 5 years. The clinical Stages of those eight patients were as follows: cStage I, 1; cII, 3; and cIII, 4. There was no significant difference between the RFS curves of “p-” and “yp-” between each Stage.

In 33 of the 51 patients with peritoneal metastasis, recurrence was diagnosed on the basis of CT findings. Twenty-one (63.6 %) of these 33 patients underwent chemotherapy after a recurrence of the disease was found. The median SAR and OS in these 33 patients were 5.3 and 20.2 months, respectively. In contrast, eight (88.9 %) of the nine patients diagnosed with peritoneal recurrence on the basis of probe-laparoscopic findings underwent chemotherapy, resulted in median SAR and OS of 12.0 and 29.6 months, respectively. The SAR of patients with peritoneal recurrence detected by probe laparoscopy was significantly longer than that detected by CT (*p* = 0.010). There was no significant difference between the OS of patients with peritoneal recurrence detected by probe laparoscopy and that detected by CT (*p* = 0.142).

### Risk factors for peritoneal recurrence

**Fig. 4:**
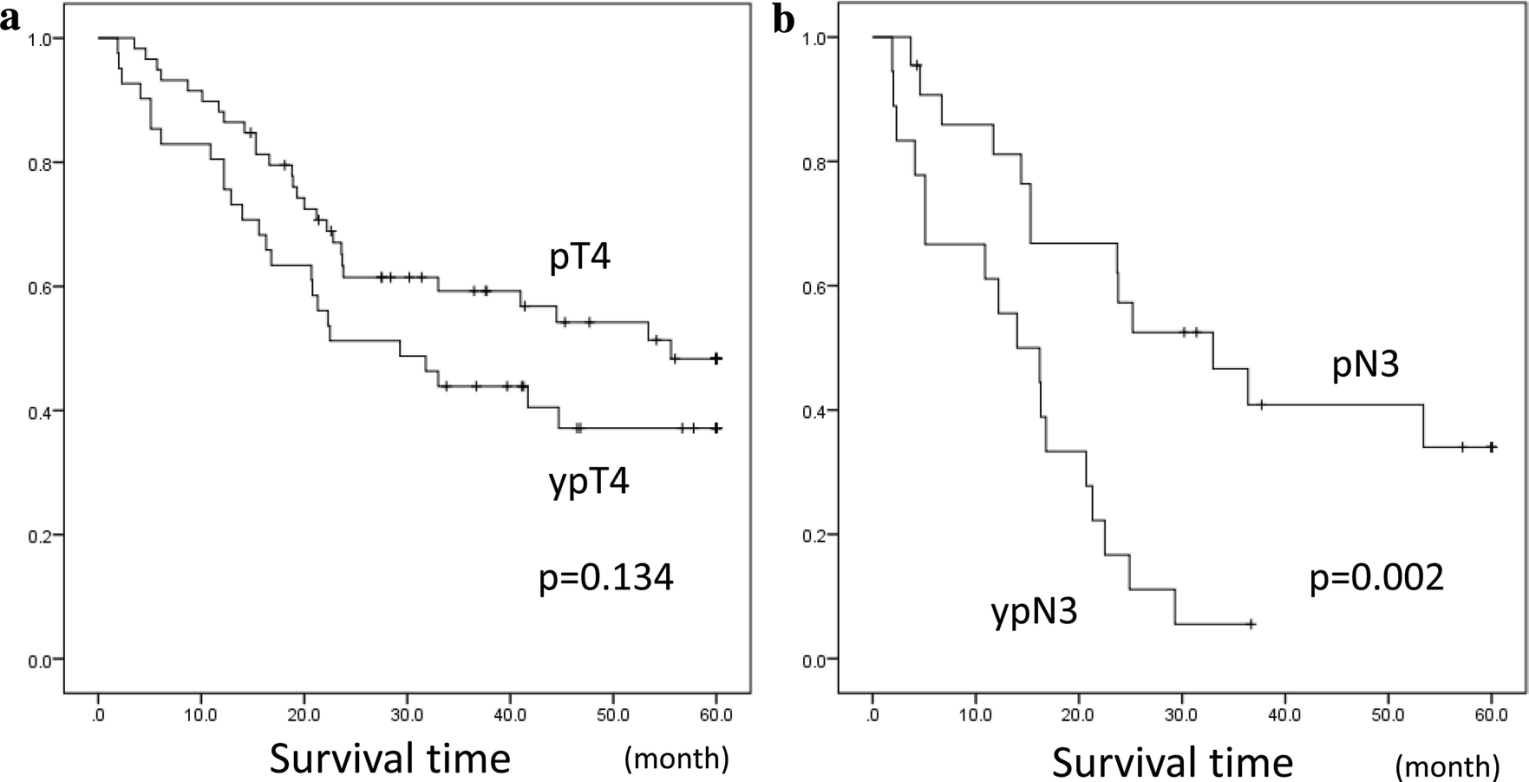
Peritoneal recurrence-free survival curves according to yp/pT4 and yp/pN. **a** Comparison of peritoneal recurrence-free survival curves according to ypT4 and pT4. **b** Comparison of peritoneal recurrence-free survival curves according to ypN3 and pN3

Multivariate analyses demonstrated that age [odds ratio (OR), 1.709; 95 % confidence interval (CI), 1.075–2.715; *p* = 0.023], vascular invasion (OR 2.134; 95 % CI 1.227–3.714; *p* = 0.007), a tumor size ≥70 mm (Aoyama et al. 2012) (OR 1.796; 95 % CI 1.084–2.975; *p* = 0.023), undifferentiated histological tumor type (OR 2.057; 95 % CI 1.242–3.404; *p* = 0.005), use of preoperative chemotherapy (OR 1.789; 95 % CI 1.137–2.814; *p* = 0.012), yp/pT4 (OR 2.595; 95 % CI 1.545–4.359; *p* < 0.001), and yp/pN3 (OR 4.113; 95 % CI 2.434–6.947; *p* < 0.001) were independent risk factors associated with peritoneal recurrence (Table 4). Peritoneal recurrence-free survival curves according to yp/pT4 and yp/pN3 are shown in Fig. 4. A significant difference was observed between peritoneal recurrence-free survival curves of ypN3 and pN3 (*p* = 0.002).

**Table 4:** Risk factors for peritoneal recurrence

Variables	Category	Multivariate		
		OR	95 % CI	*p* value
N factor	yp/pN3	4.113	2.434–6.947	<0.001
T factor	yp/pT4	2.595	1.545–4.359	<0.001
Histological type	Undifferentiated	2.057	1.242–3.404	0.005
Vascular invasion	Positive	2.134	1.227–3.714	0.007
Tumor size	>70 mm	1.796	1.084–2975	0.023
Preoperative chemotherapy	Yes	1.789	1.137–2.814	0.012
Age	≥65 y.o.	1.709	1.075–2.715	0.023

*CI* confidence interval, *OR* odds ratio

## Discussion

In this study, we analyzed long-term outcomes and recurrence patterns in patients who underwent an R0 resection using LG-D2 for cStage ≥ IB gastric cancer. The 5-year survival rates stratified by yp/pStage in this study were slightly lower than those in previous LG studies (Kim et al. 2010), this may be partly because 128 patients (36.2 %) who underwent PC were included in this study. The 5-year survival rate following a total gastrectomy has been shown to be lower than that following a distal gastrectomy (Maruyama et al. 1991; Isobe et al. 2011) at least partly because a total gastrectomy is more commonly performed in patients with large gastric cancer (Im et al. 2012) than distal gastrectomy. Actually, in this study, the proportion of patients who underwent LTG out of all patients who underwent LG was 28.9 %, which is higher than that in other previous LG reports, (<10 %) (Kim et al. 2010, 2012) and is comparable with that mentioned in OG reports (20–40 %) (Maruyama et al. 1991; Isobe et al. 2011; Sakuramoto et al. 2007; Sasako et al. 2008).

Regarding the proportion of initial recurrence patterns, on the one hand, there was a considerable difference in locoregional lymph node metastasis and local recurrence between this study (0 %) and previous studies (present study versus previous studies: locoregional lymph node metastasis, 0 versus 8–22 %; local recurrence 0 versus 8–54 %) (Sasako et al. 2008; Lee et al. 2014; D’Angelica et al. 2004; Tajima et al. 2004), suggesting that the quality of our LG-D2 is acceptable. On the other hand, the frequency of peritoneal metastasis was remarkably higher (present study vs. previous studies: 59.3 vs. 29–38 %). This may be partly because we readily performed probe laparoscopy to determine the presence of a peritoneal recurrence once a tumor recurrence was suspected (e.g., elevation of serum tumor marker levels, despite the absence of overt recurrence in imaging findings). The use of probe laparoscopy to observe the abdominal cavity following LG must be easier than that following OG since adhesions in the abdominal cavity after LG are less severe. Therefore, we performed probe laparoscopy without any hesitation when a peritoneal recurrence was suspected but could not be determined via imaging studies. In fact, extensive adhesions were not observed in any patient enrolled in this study, and probe laparoscopy was safely performed in all 11 cases. Then, the site of recurrence was identified in all patients in this study, although previous studies regarding the long-term outcomes following OG demonstrated recurrence rates at 14–46 % at unknown sites (Maruyama et al. 1991; Isobe et al. 2011; Otsuji et al. 2004), indicating that a definitive diagnosis of peritoneal recurrence by imaging alone is difficult (Shim et al. 2011; Kim et al. 2009). Moreover, in this study, the SAR of a peritoneal metastasis diagnosed by probe laparoscopy was extended compared with that diagnosed by CT. Thus, under excellent local control via LG-D2, peritoneal recurrence is expected to be an overwhelmingly dominant recurrence pattern as long as peritoneal metastasis is the most common cause of death in gastric cancer patients; this suggests that the active use of probe laparoscopy potentially promotes an accelerated detection of tumor recurrence, particularly occult tiny peritoneal metastasis. Further study is warranted to verify whether early detection of tumor recurrence leads to an improvement in long term outcomes or not.

In this study, the peritoneal recurrence rate increased concurrently with increases in yp/pStage. Actually, in the present study, the peritoneal recurrence rates were 39 % in cases of yp/pT4 disease and 52 % in cases of yp/pN3 disease, which were comparable with those reported in the previous OG studies (43.7–44.9 % for T4 and 37.4–54.0 % for N3) (Aoyama et al. 2012; D’Angelica et al. 2004; Tajima et al. 2004; Lee et al. 2014). In addition, in this study, a multivariate analysis demonstrated that T4, N3, a tumor size ≥70 mm, vascular invasion, and undifferentiated tumor type were independent risk factors for peritoneal metastasis following LG-D2. Similar results have been reported in previous OG studies (Aoyama et al. 2012; Lee et al. 2014), which reported that a tumor size ≥70 mm, T4, N3, and vascular invasion were predictive factors for peritoneal metastasis following OG. Thus, the incidence and predictors of peritoneal metastasis after LG-D2 appear to be comparable with those of peritoneal metastasis after OG.

It was possible that a negative conversion from P1 or Cy1 to P0 or Cy0 occurred because of PC and remained undetected because a staging laparoscopy was not performed for all patients before PC in this study. Thus, Stage IV gastric cancer could have potentially migrated to other ypStages after PC. This may be one of the reasons for the relatively high rate of peritoneal recurrence compared with other recurrence patterns in this study. Furthermore, the peritoneal recurrence-free survival curve of ypN3 was significantly inferior compared with that of pN3. This suggested that ypN3 patients need careful follow up for peritoneal recurrence particularly.

There are some limitations to this study. First, this study was conducted in a retrospective manner. Second, the sample size was relatively small. Third, use of preoperative chemotherapy may have positively or negatively affected OS and RFS since the beginning date of OS and RFS was defined as that of the initial surgery but not the initial diagnosis.

In conclusion, at least under good LG-D2 local control, the oncological effect of LG-D2 was comparable with that of OG. As long as the recurrence occurs mostly on the peritoneum following LG-D2, probe laparoscopy, which is compatible with the status after LG causing less extensive intraabdominal adhesion, should be actively used to detect minimal peritoneal recurrence after LG-D2.

## Abbreviations

LG-D2: laparoscopic gastrectomy with D2 lymphadenectomy
LDG: laparoscopic distal gastrectomy
ODG: open distal gastrectomy
LN: lymph node
LTG: laparoscopic total gastrectomy
D2 + PS: D2 lymphadenectomy combined with a distal pancreaticosplenectomy
D2 + S: D2 lymphadenectomy combined with a splenectomy
D2-S: a spleen-preserving D2 lymphadenectomy
D2-10: D2 lymphadenectomy with preservation of station 10 LNs, and the spleen
PC: preoperative chemotherapy
CT: computed tomography
US: ultrasonography
MRI: magnetic resonance imaging
PET-CT: positron emission tomography-CT
RFS: recurrence-free survival
SAR: survival after recurrence
OS: overall survival
OR: odds ratio
CI: confidence interval
